# Mining biomarker information in biomedical literature

**DOI:** 10.1186/1472-6947-12-148

**Published:** 2012-12-18

**Authors:** Erfan Younesi, Luca Toldo, Bernd Müller, Christoph M Friedrich, Natalia Novac, Alexander Scheer, Martin Hofmann-Apitius, Juliane Fluck

**Affiliations:** 1 Department of Bioinformatics, Fraunhofer Institute for Algorithms and Scientific Computing (SCAI), Schloss Birlinghoven, Sankt Augustin, 53754, Germany; 2 Bonn-Aachen International Center for Information Technology (B-IT), University of Bonn, Bonn, Germany; 3 Knowledge Management, Operational Excellence & Site Coordination, Merck Serono, Merck KGaA, Darmstadt, Germany; 4 Informatics & Knowledge Management, Merck Serono, Merck KGaA, Geneva, Switzerland; 5 Department of Computer Science, University of Applied Science and Arts, Dortmund, Germany

**Keywords:** Text-mining, Biomarker discovery, Information retrieval, Terminology

## Abstract

**Background:**

For selection and evaluation of potential biomarkers, inclusion of already published information is of utmost importance. In spite of significant advancements in text- and data-mining techniques, the vast knowledge space of biomarkers in biomedical text has remained unexplored. Existing named entity recognition approaches are not sufficiently selective for the retrieval of biomarker information from the literature. The purpose of this study was to identify textual features that enhance the effectiveness of biomarker information retrieval for different indication areas and diverse end user perspectives.

**Methods:**

A biomarker terminology was created and further organized into six concept classes. Performance of this terminology was optimized towards balanced selectivity and specificity. The information retrieval performance using the biomarker terminology was evaluated based on various combinations of the terminology's six classes. Further validation of these results was performed on two independent corpora representing two different neurodegenerative diseases.

**Results:**

The current state of the biomarker terminology contains 119 entity classes supported by 1890 different synonyms. The result of information retrieval shows improved retrieval rate of informative abstracts, which is achieved by including clinical management terms and evidence of gene/protein alterations (e.g. gene/protein expression status or certain polymorphisms) in combination with disease and gene name recognition. When additional filtering through other classes (e.g. diagnostic or prognostic methods) is applied, the typical high number of unspecific search results is significantly reduced. The evaluation results suggest that this approach enables the automated identification of biomarker information in the literature. A demo version of the search engine SCAIView, including the biomarker retrieval, is made available to the public through http://www.scaiview.com/scaiview-academia.html.

**Conclusions:**

The approach presented in this paper demonstrates that using a dedicated biomarker terminology for automated analysis of the scientific literature maybe helpful as an aid to finding biomarker information in text. Successful extraction of candidate biomarkers information from published resources can be considered as the first step towards developing novel hypotheses. These hypotheses will be valuable for the early decision-making in the drug discovery and development process.

## Background

During the past years, high-throughput technologies have been extensively employed for the study of molecular mechanisms underlying different diseases; this has led to the discovery of a large number of molecular biomarkers [1]. The US National Institutes of Health defines a biomarker as “a characteristic that is objectively measured and evaluated as an indicator of normal biological processes, pathogenic processes or pharmacological responses to a therapeutic intervention” [2].

Depending on representing molecular, physiological, or structural features, biomarkers show significant diversity – spanning from genes, proteins, DNA, RNA, and SNPs to blood cholesterol levels and patterns of brain abnormality. Due to such diverse coverage, ambiguity in defining biomarkers types and classes continues to exist [3-5].

Application of biomarkers, however, goes beyond disease prediction and monitoring; in fact they have also been utilized throughout various stages of drug discovery and development [6]. For example, biomarkers play an important role in drug target discovery and validation (e.g. as quantitative readouts for candidate drugs) [7], in the monitoring of toxicity mechanisms (e.g. assessment of indication of unwanted side-effects) [8], and non-invasive imaging of diseased organs [9]. In the process of drug development, biomarkers are considered to be pivotal for informed go/no-go decision-making in the early stages of drug development [10]. For example, mechanistic biomarkers can be used to pre-clinically measure a drug's pharmacological activity in terms of its distribution and interaction with a defined protein target. Such measurements help to decide whether to move forward to the next phase of clinical development.

A first step to finding supportive evidence for clinically important potential biomarkers is to search the accumulated data and knowledge generated from basic research [11]. For efficient exploration of the suspected large amount of biomarker information contained in the biomedical literature, semantic search and information retrieval systems are of utmost importance. The largest publicly available biomedical literature resource, PubMed, makes use of MeSH (Medical Subject Headings) concepts to annotate the abstracts so that it maps these concepts to user queries and by this means allows for semantic search [12]. However, to search for reported biomarkers, (e.g. genes, proteins, or genetic variations) in a text corpus, additional annotation of these entities and their normalization to relevant databases is required. Such a search is currently not provided by PubMed. Manual annotation is also time consuming and lags behind due to the ever abundance of new publications. For example, compiling a compendium of potential biomarkers for pancreatic cancer was carried out by systematic manual curation of the literature and took over 7,000 person hours [13].

The automated identification of relevant terminology, a process known as Named Entity Recognition (NER), can support semantic annotation and information retrieval. In the past years, several NER methods for the extraction of different biological entities have been developed, primarily focusing on the recognition of gene and protein names. In this regard, the BioCreAtIvE assessments present an overview about the state of various technologies and approaches in use [14-16]. NER approaches have already been used to support the identification of biomarker genes. In [17], the authors applied gene and protein name recognition techniques to Medline and OMIM (Online Mendelian Inheritance in Man) to identify potential serum biomarkers for Down syndrome. Similarly, a named entity tagging approach was employed to search for prostate cancer biomarker candidate genes in OMIM records [18]. Studies on biomarker relation extraction from text have considered the extraction of semantic relations between diseases and genes or proteins [19,20]. In a recent study, an information extraction framework has been developed for the classification of biomarker sentences that showed favourable results, but the study was unfortunately focused on a small training set [21]. Moreover, biomarker information is often dispersed over the entire abstract, making it difficult to reach high information recall with sentence classification systems.

The aim of this work is to analyze how information about potential biomarkers is expressed in the complete body of a scientific text, and identify additional textual features that are relevant for the retrieval of potential biomarker information from the scientific literature.

## Methods

### Information retrieval system and entity ranking

**Figure 1:**
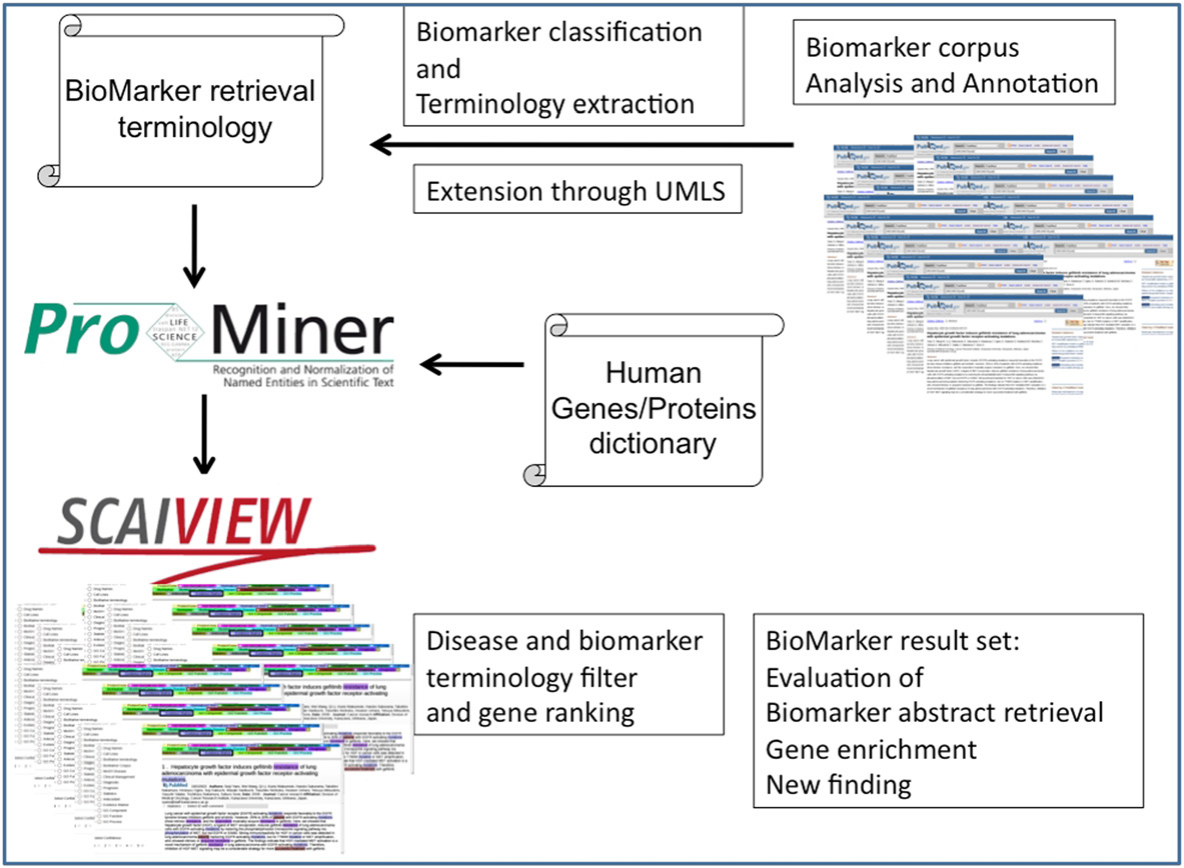
An overview of the biomarker retrieval system design.

Our retrieval system is composed of two software components: the named entity recognition tool ProMiner and the knowledge discovery framework SCAIView [22,23]. Figure 1 shows the design overview of the biomarker information retrieval system. ProMiner is one of the systems for gene normalization, which performed very well in BioCreAtIvE I and II assessments [24-26], reaching an F-score of 0.8 for the recognition and normalization of human genes and proteins. The ProMiner system has been designed for the semi-automated generation of dictionaries and the recognition of spelling variants, as well as the disambiguation of acronyms and common word synonyms.

**Figure 2:**
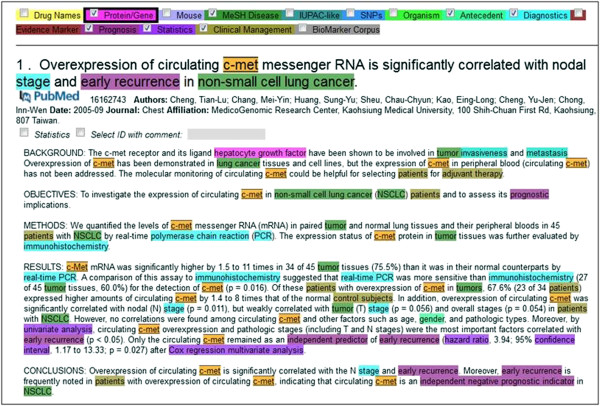
Example annotation of a biomarker abstract in SCAIView.

The biomarker retrieval terminology and other dictionaries were incorporated into ProMiner, followed by ProMiner's annotation of all Medline abstracts using the dictionaries. The resulting entity annotations are stored in an Apache Lucene(TM)-based search index, together with the documents and their meta-information. SCAIView incorporates the Lucene-based index and allows for searches that include exact matches, wildcard options, and Boolean operations. The document visualization within SCAIView provides highlighting for all the entity classes with tooltips containing available linkouts, descriptions, and depictions (Figure 2).

**Figure 3:**
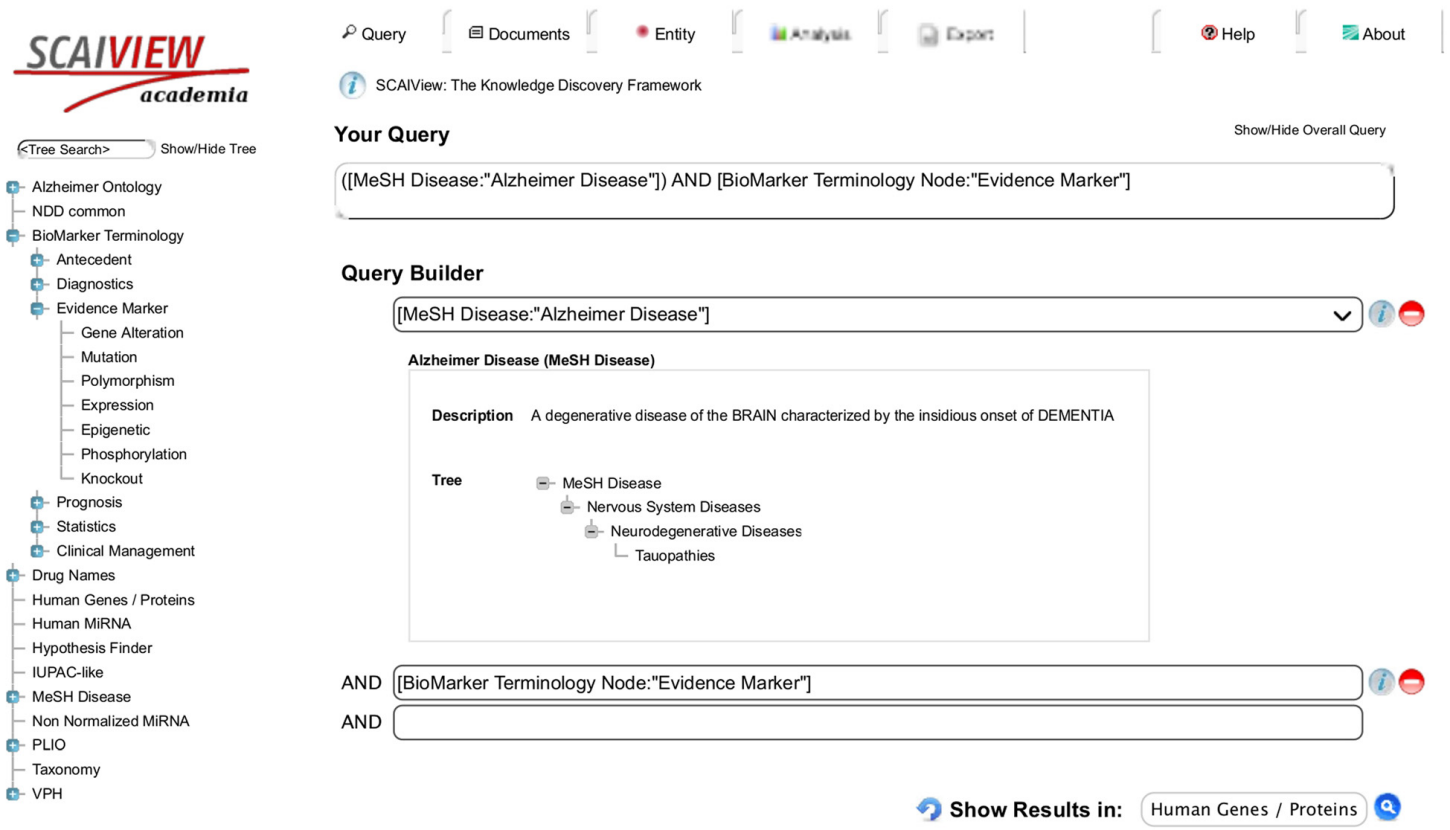
**Incorporation and usage of biomarker terminology in SCAIView.** Biomarker terminology is visualized in SCAIView as a navigable tree (left). The user query is formulated either by auto-complete finding of the concepts in Query Builder or navigating the tree. Finally, the type of output (entities or documents) is selected from the drop-down menu.

The annotations are organized in the form of hierarchies (semantic trees) and can be navigated by the user via selecting full classes (e.g. ‘genes’ or ‘diseases’), selecting certain subclasses (e.g. ‘Nervous Systems Diseases’), or singular dictionary entries (e.g. ‘Alzheimer’s Disease’). For Boolean operations, either single entity class or complete tree classes could be selected. For example, the query “ ‘Alzheimer’s Disease’ AND ‘Evidence marker’ ” would retrieve all abstracts that contain one or more terms of both terminology classes. By selecting the *Human Genes/Proteins*, an entity table containing a list of the recognized genes and proteins in the selected corpus would be displayed (Figure 3).

The biomedical entities are ranked either by frequency or by relative entropy (in descending order of importance). The frequency ranking orders the entities with respect to the number of citations. The relative entropy ranking is based on the Kullback–Leibler divergence (also known as information gain) [27,28], and calculates the fraction between the documents containing the entity in the result set and the total number of documents containing the entity in the complete Medline document set. The formula for the relative entropy is given as:

(1) $$RE\left(p_{1},p_{2}\right)=p_{1}\cdot \;\log\frac{p_{1}}{p_{2}}=p_{1}\cdot \;\left(\text{log}p_{1}-\text{log}p_{2}\right)$$

where p_1_ is the number of abstracts containing the entity in the query selected corpus and p_2_ denotes the total number of documents in which the entity occurs within an unspecific reference corpus (i.e. the entire Medline). The Kullback–Leibler divergence ranks those entities high, which have especially high frequency in the selected corpus in comparison to the unspecific reference corpus. This means that frequently occurring entities do not receive high ranks. For example, using the query “ ‘Alzheimer’s Disease’ AND ‘Evidence marker’ AND ‘Human Genes/Proteins’ ”, we retrieved 331 abstracts containing IL1B with a frequency ranking of 10. Conversely, according to the relative entropy formula, IL1B has an entropy rank of 34 despite its high occurrence in Medline (i.e. 40685 abstracts).

### Corpus annotation and terminology generation

Five end users were asked to provide 50 relevant abstracts containing cancer or drug-related biomarker information. The abstracts were chosen from the following areas due to data availability and their current interest in treating cancer.

NSCLC (Non-Small Cell Lung Cancer) related, Breast cancer and predictive/prognostic, Met signaling and cancer, EGFR related, Gefitinib related, and Erbitux related.

A training corpus of 289 abstracts was established (see Additional file 1 for PMIDs of the training abstracts), and two annotators (different from the end users) were asked to annotate 10 abstracts out of this corpus in a first step. The annotators manually marked all biomarker information evidence that were perceived to be relevant (see Figure 2 for an example of automatic annotations). Instead of annotating the biomarkers themselves (i.e. the gene, protein, or SNP mentions), the biomarker evidence was annotated and assigned to six classes. These classes were chosen based on the conventional classification of biomarker types as well as the distribution analysis of the potential biomarker information in the literature. Subsequently, the following simple annotation guideline was generated:

Clinical Management: annotate all terms indicating clinical investigations in patients, which includes the initial mentioning, the clinical study, and finally the treatment

Diagnostics: annotate all diagnostics that are used, which includes the initial disease stage, the molecular identification, and blood diagnostics

Prognosis: annotate all terms indicating any prognosis, outcome, or marker (e.g. clinical or biomarkers, adverse effects, resistance, response, disease progression or outcome)

Evidence marker: annotate all changes in gene and protein abundance, spanning from expression to mutation, SNP variations to phosphorylation status

Antecedent: annotate all risk factors mentioned for the relevant disease

Using this guideline, both annotators annotated 50 abstracts independently, and resulting agreement between them was measured based on a kappa value. A kappa value measures the degree of confidence between the two annotators as follows [29]:

(2) $$\text{kappa}=\frac{\left(\mathrm{Pr}\left(a\right)-\mathrm{Pr}\left(e\right)\right)}{\left(1-\mathrm{Pr}\left(e\right)\right)}$$

where Pr(a) is the relative observed agreement among the raters and Pr(e) is the probability of random agreement. The remaining abstracts were then annotated by one of the annotators.

### Performance evaluation

For all evaluations, our search for Alzheimer’s disease (AD) or multiple sclerosis (MS) scenarios was expanded using MeSH synonyms and matches of the human gene/protein dictionary. The ranked gene list provided by SCAIView was compared with the gold standard gene set that we obtained for AD and MS from the BIOBASE database [30]. This database contains manually curated literature evidence for causal and correlative associations of genes and proteins to human diseases. Both gold standards were created by filtering property reports, based on the disease indication, and then importing the resulting list of genes and proteins to a text file based on their supporting evidence (i.e. experimentally determined). To evaluate the performance, a gene enrichment ranking methodology adopted from the Gene Set Enrichment Analysis (GSEA) was applied [31]. The goal of the enrichment is to determine whether genes within the gold standard (gene set S) are randomly distributed throughout the ranked list of retrieved genes L (with length N) or primarily found at the top or bottom of the list. Computed score is the maximum deviation from zero when walking down the list of genes L that is increased by X_j_ =1 every time a gene in the list L is in S. Thus, the enrichment score (ES) is calculated as:

(3) $$\mathrm{ES}=\underset{1\leq i\leq N}{\text{max}}\sum_{j=1}^{i}X_{j}$$

Knowing that the output of the retrieval system is a list of genes/proteins that are ranked according to their relative entropy scoring, different combinations of the biomarker terminology classes were applied. For all combinations, values of precision, recall and F-score were calculated. In order to compare the ranked gene list with the gold standard, the F-score criterion was used. An F-score is computed as the following:

(4) $$F-\mathrm{score}=\frac{2\times \text{Precision}\times \text{Recall}}{\left(\text{Precision}+\text{Recall}\right)}$$

where Precision is defined as TP/(TP+FP) and Recall is defined as TP/(TP+FN) [32], and TP = True Positive, FP = False positive, TN = True Negative (TN) and FN = False Negative (FN).

From the list of retrieved entities, eleven top ranking genes/proteins were selected for an analysis, assessing how much biomarker information content is contained in their corresponding abstracts. Since more than one abstract is retrieved per gene and protein, and because a typical user might base its decision on the first retrieved abstracts (the most recent publications), only the first ten abstracts were taken into account. Subsequently, and after manually checking of abstracts, the number of true positive and false positive biomarker abstracts was determined.

## Results

### Development of biomarker retrieval terminology

The training corpus containing cancer or drug-related biomarker information was pre-annotated using gene protein and disease named entity recognition. In the next step, we investigated if we could identify additional terminology relevant to biomarkers from the abstracts. Initially two annotators highlighted relevant parts in 10 abstracts and discussed the annotations. As a result, six terminology classes were defined representing the relevant content for biomarker identification (Table 1). Abstracts containing information about potential gene biomarkers most often comprise *evidence* of gene or protein alteration as well as indication words for the *clinical management* or investigation. Moreover, *diagnostic* methods and *prognostic* terms are found frequently in these abstracts. *Statistic* evaluations strengthen the relevance of biomarkers and in some cases *antecedents* are strong indicators for disease status or therapy decisions (see Figure 2). Based on those terminology classes and a simple annotation guideline, all 10 abstracts were again annotated and discrepancies were discussed until a common understanding was reached between annotators. In the next step, the two annotators annotated 50 abstracts and the inter-annotator agreement was calculated. The kappa value of 0.81 showed that both annotators were in good agreement when annotating concepts represented by the six classes. Inconsistencies were mainly because of missed annotations (e.g. the concept ‘patient’ was annotated 3 times instead of 4 times in one abstract), boundary differences (e.g. annotation of ‘management of patients’ versus annotation of ‘patients’) and some differences in the annotation of diagnostic terms (e.g. the concept ‘Child’s score’ was only annotated by one annotator). Those differences were discussed and one annotator annotated the remaining abstracts.

**Table 1:** Biomarker retrieval terminology classes and coverage of the terminology in the annotated corpora

**Terminology class**	**Description**	**Examples**	**Annotation found in abstracts**
Clinical Management	Terms indicating clinical investigations on patients	Patient; Cohort study	96%
Diagnostics	Terms representing clinical as well as molecular diagnostics	Immunohistochemistry; Emission Tomography; Microarray	85%
Prognosis	Terms indicating the prediction for a patient /kind of biomarker/ outcome of therapies	Surrogate end point; clinical response; biomarker; predictor	88%
Statistics	Statistical methods indicating the strength of the biomarker relationship	Chi(2) test; mean +/− SD; univariate analysis; Kaplan-Meier Analysis	48%
Evidence	Terms describing genetic/ molecular evidence for activity of a gene	Mutation; gene amplification; polymorphism; expression	82%
Antecedent	Terms expressing exposure to hazardous agents and risk factors	Smoker; susceptibility; exposure	20%

All annotated terms in the training corpus were automatically extracted and integrated into a seed biomarker retrieval dictionary by manually assigning them to the six main classes. In the next step, the seed set was structured, forming a seed terminology containing 76 entity classes with 1132 different synonyms. After extension of the terminology with similar classes and synonyms from MeSH and UMLS as well as expert knowledge for diagnostic tests (after the analysis of larger disease- and biomarker-related text corpora), the biomarker retrieval terminology contained 119 entity classes with 1890 different synonyms.

Distribution analysis of different biomarker classes in relevant text corpora (annotations in abstracts) showed that every training abstract contains at least one biomarker retrieval term. Moreover, *Clinical Management* and *Prognosis* terms were found most frequently in the corpora, followed by *Diagnostic*, *Evidence* and *Statistics* terms (see Table 1, last column). The *Antecedent* class plays a minor role in the selected training data (recall of 20%), which might be quite different for diseases in which antecedents play a role in biomarker search. The six terminology classes are integrated into the SCAIView Demo server and can be accessed through http://www.scaiview.com/scaiview-academia.html.

### Non-small cell lung carcinoma (NSCLC) as use case scenario

As a base line for biomarker candidate retrieval, selection of the disease NSCLC in combination with the automated recognition of gene names in our retrieval system returns 10790 Medline abstracts mentioning at least one gene concept (2663 different gene entities in total). Without further specification, these numbers are far too high to inspect and the resulting abstracts and genes cover various aspects of NSCLC-gene relations not relevant for the search of NSCLC biomarker candidates. Putting additional restriction on the semantic search using the five biomarker retrieval classes (ignoring the class Antecedent) reduces the number of retrieved documents down to 1539, containing 877 genes. Further reduction to relevant genes is possible through the inclusion of frequency or relative entropy ranking thresholds. Ignoring all gene mentions occurring in less than 5 abstracts decreases the gene set to 126 genes. Spot checks of ten genes with low relative entropy values revealed that nine of these genes are true positive biomarker candidates (see Table 2).

**Table 2:** Spot check of NSCLC genes/proteins for their relevance to biomarker applicability and evidence

**Gene or protein**	**No. of retrieved document**	**Relative entropy rank**	**Frequency based rank**	**Example PMID**	**Biomarker applicability**	**Biomarker evidence**
P53	281	1	1	20521348	Prognosis	Expression
VEGFA	128	6	4	21964530	Prognosis	Expression
BCL2	90	8	6	19560836	Survival	Expression
PCNA	47	11	10	21495034	Prognosis	Expression
VEGFC	22	16	21	19758816	Recurrence	Expression
PTGS2	33	25	15	20592629	Survival	Expression
CDKN1B	12	50	52	15483027	Prognosis	Expression
DNAJB4	2	100	344	16788156	Survival	Expression
CDK2	5	342	112	10817513	Prognosis	No impact

For the SCAIView search the MeSH disease “Carcinoma, Non-Small-Cell Lung” in combination with five biomarker retrieval classes (omitting the class Antecedent) were selected and the retrieved genes were ranked according the Relative Entropy or frequency. In spot tests the abstracts were analysed for containing biomarker information for the respective genes.

One of the biomarker candidates is Mucin 1 for which 8 abstracts under the above- mentioned criteria were retrieved. Mucin 1 is involved in invasiveness, metastasis, and angiogenesis of NSCLC [33] and its expression and localisation in lung adenocarcinoma patients is altered compared to normal epithelial cells [34]. A further exemplified action using the biomarker retrieval terminology is the literature search for suitable Mucin-1 antibodies. This can be directly conducted by applying the biomarker retrieval terminology. Selection of the *Diagnostic* subclass *Immunohistochemistry* and the *Evidence* subclass *Expression* together with the NSLCL MeSH variants and the Mucin-1 gene concept retrieves 16 abstracts. In 11 abstracts immunostaining of Muc1 is directly stated but only 4 abstracts mention the specific antibodies (CA15-3, clone DF3 in PMID 17409826, HMFG2 in PMIDs 8980247, 2456176 and 1284790). A further analysis of the retrieved full text articles is necessary to retrieve in a next step all information about the antibodies utilized. Higher recall rates for different antibodies for this example could be reached with searches for a broader disease area like *Lung Neoplasms* (31 abstracts), the search with the *Diagnostic* subclass *Immunohistochemistry* in combination with the text search ‘anti-Muc1’ (105 abstracts), or in combination with the gene concept *Muc1* and the Evidence marker *Expression* (1055 abstracts).

These examples demonstrate the ability of the biomarker search engine - depending on the application area - to substantially increase recall or precision of the search results, maximizing the efficiency of public domain literature searches.

### Adaptation and evaluation of biomarker retrieval in neurodegenerative diseases area

To demonstrate the applicability of the biomarker retrieval terminology in other disease indications independently of the oncology-focused training corpus, the performance of retrieval for biomarker-related abstracts from Medline was tested in the field of Alzheimer’s disease and multiple sclerosis.

In a first test it became evident that the class *Diagnostics* shows a high diversity between cancer and neurodegenerative diseases and that this class has to be extended for the new disease areas. For instance, in the area of neurodegenerative diseases, we included the diagnostic imaging subtree of MeSH (after omitting microscopy and molecular imaging) and a number of very specific medical classification systems provided by experts (e.g. MS Functional Composite score). The final BioMarker terminology contains 164 classes and 2506 synonyms.

After this optimization for neurodegenerative diseases, in three evaluation steps we examined: i) to what extent biomarker class selection influences the biomarker gene enrichment in comparison to the content of independent gold standards (entity retrieval); ii) how many biomarker-enriched abstracts could be retrieved with the selection of our terminology (abstract retrieval); and iii) how far new biomarker genes/proteins information could be retrieved which are not provided by the gold standard (potential biomarker identification).

**Figure 4:**
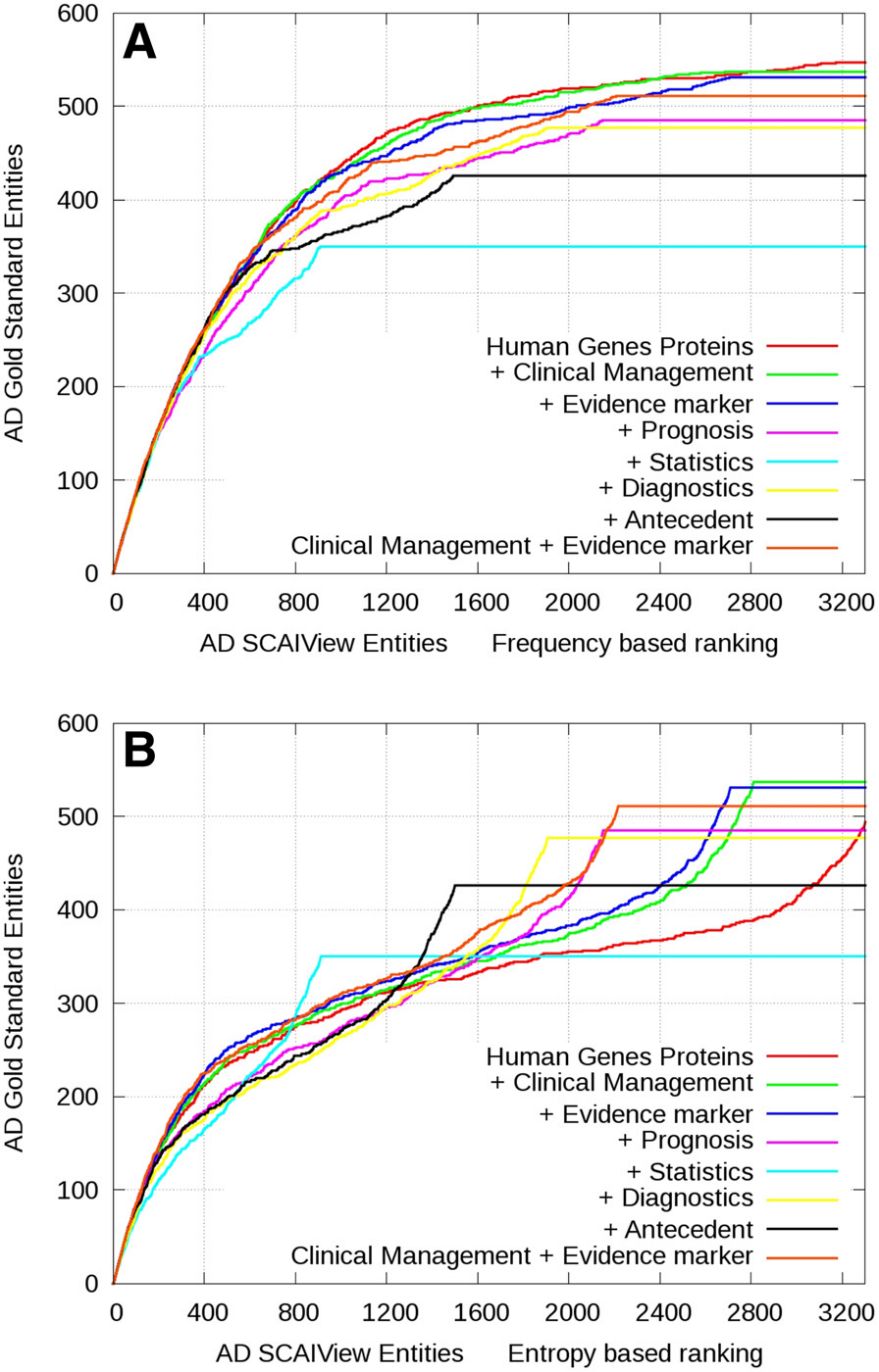
**Comparative gene enrichment plots for gene/protein entities retrieved in the context of AD.** Genes were ranked based on frequency (**A**) or relative entropy (**B**) and evaluated against the Alzheimer’s genes/proteins gold standard. The red color represents the retrieval of all abstracts containing human genes/proteins. The other color codings indicate the retrieval rate after additive inclusion of further terminology classes.

**Figure 5:**
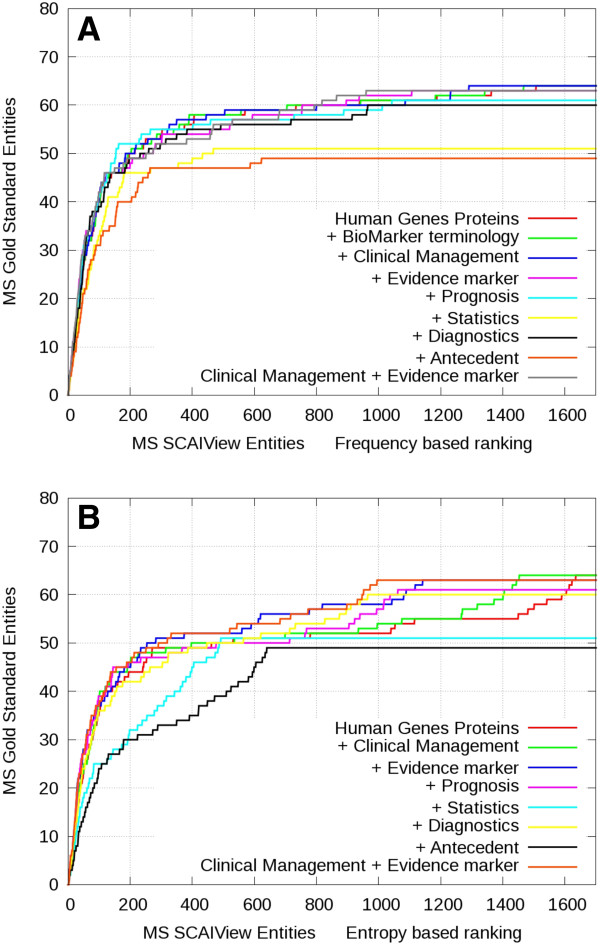
**Comparative gene enrichment plots for gene/protein entities retrieved in the context of MS.** Genes were ranked based on frequency (**A**) or relative entropy (**B**) and evaluated against the multiple sclerosis genes/proteins gold standard. The red color represents the retrieval of all abstracts containing human genes/proteins. The other color codings indicate the retrieval rate after additive inclusion of further terminology classes.

Using the gene enrichment ranking method and the selection of *Human Genes/Proteins* (our baseline approach), it turns out that almost all genes/proteins contained in the gold standard (95% for Alzheimer’s and 97% for multiple sclerosis) were covered by the results of our retrieval system (see Human Genes/Proteins in Figures 4 and 5).

The system was able to successfully extract candidate biomarker genes/proteins relevant to the queried diseases and both ranking methods performed well for high-ranking genes. Nevertheless, the baseline search returned over 3600 genes from more than 33000 abstracts for Alzheimer’s disease and over 2300 genes from more than 12900 abstracts for multiple sclerosis. This amount of abstracts for manual inspection is overwhelming. By additive selection of the *Clinical Management* or *Evidence Marker* classes, it can be shown that the relevant biomarker candidate genes are retrieved at higher ranks in both Alzheimer’s and multiple sclerosis contexts (Figure 4 and Figure 5). For all other classes, the slope of gene enrichment decreases at lower ranks in comparison to selection of *Human Genes/Proteins* alone. Overall, frequency-based ranking (Figure 4A and Figure 5A) seems to perform better than relative entropy-based ranking (Figure 4B and Figure 5B). Although frequency-based ranking leads to higher gene enrichment slope between the ranks 300 and 1500, we observe a stronger enrichment at low ranking positions using relative entropy-based ranking. Analysis of those low ranking but frequent genes in Alzheimer’s disease and multiple sclerosis shows that the high number of cytokines and inflammatory proteins are mere indicators of the disease. The frequency of those genes in the whole Medline is very high and for this reason they are underrepresented in the specific corpus.

**Figure 6:**
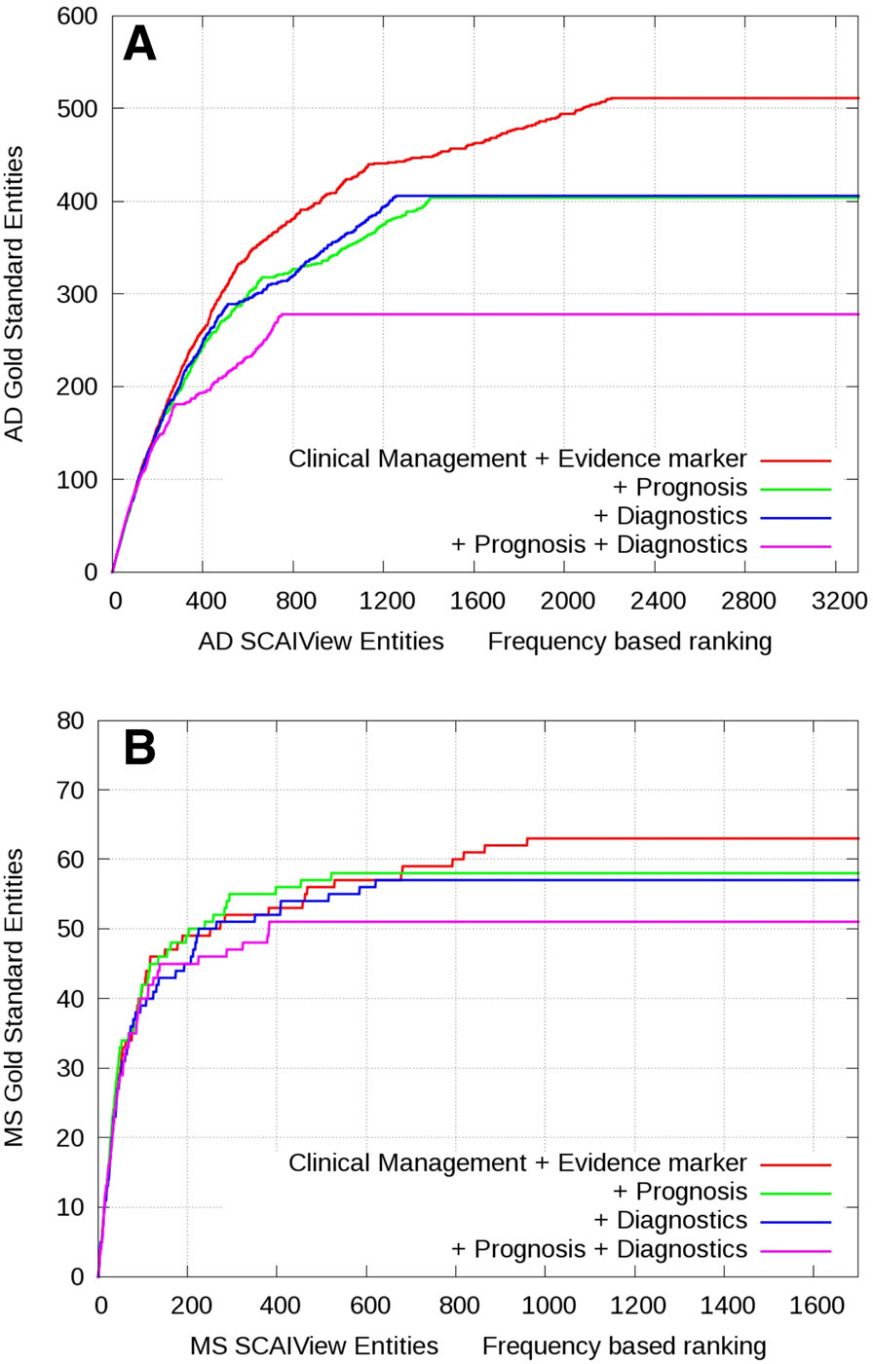
**Frequency-based gene enrichment plots after combining additive classes in the context of AD and MS.** Genes were ranked based on frequency and evaluated against the Alzheimer’s (**A**) or multiple sclerosis (**B**) gold standards. The color codings indicate the retrieval rate after additive inclusion of further terminology classes.

Using an AND combination of three or more terminology classes leads to similar enrichment slopes for higher ranks but with reduced recall (Figure 6). When compared to the MS gold standard, the combination of classes *Clinical Management*, *Evidence Marker* and *Prognosis* or *Diagnosis* performs even better than *Clinical Management* and *Evidence Marker* alone but loses some recall at lower ranks.

For Alzheimer’s disease, we analyzed the outcome of the frequency based ranking method and calculated the precision, F-score and rank for different recall ranges (10%, 30%, and 50% recall) and determined the maximal F-score (see Table 3). For the classes *Clinical Management* and *Evidence Marker* as well as for the combined selection of both classes, the values are similar but the maximal F-score (0.59 for *Genes/Proteins* alone and 0.58 for the combination) is reached at lower ranking positions (rank 555 for the combination instead of rank 728 for Genes/Proteins alone). For recall value of 0.3, the precision is similar for all selections even with the combination of the classes *Clinical Management*, *Evidence Marker* and *Prognosis*. At recall value of 0.5, the precision drops down to 0.5, otherwise 0.63 if the class Prognosis is not selected; moreover, the maximal F-score looses 7% in comparison to *Genes/Proteins* alone.

**Table 3:** Performance evaluation for Alzheimer’s disease

**Selection**	**Rank**	**Recall**	**Precision**	**F-score**
Baseline: Genes / Proteins	60	0.10	0.92	0.17
	230	0.30	0.73	0.42
	469	0.50	0.61	0.55
*Maximal F-score*	728	0.67	0.52	0.59
Baseline + Clinical Management	61	0.10	0.90	0.17
	226	0.30	0.75	0.42
	465	0.50	0.61	0.55
*Maximal F-score*	682	0.65	0.54	0.59
Baseline + Evidence Marker	62	0.10	0.89	0.17
	225	0.30	0.75	0.42
	464	0.50	0.61	0.55
*Maximal F-score*	654	0.62	0.54	0.57
Baseline +Prognosis	63	0.10	0.87	0.17
	247	0.30	0.68	0.41
	541	0.50	0.52	0.51
*Maximal F-score*	740	0.61	0.47	0.53
Baseline + Diagnostics	64	0.10	0.86	0.17
	237	0.30	0.71	0.42
	494	0.50	0.57	0.53
*Maximal F-score*	520	0.52	0.57	0.54
Baseline + Statistics	64	0.10	0.86	0.17
	227	0.30	0.74	0.42
	678	0.50	0.42	0.45
*Maximal F-score*	377	0.41	0.62	0.49
Baseline + Clinical Management + Evidence Marker	60	0.10	0.92	0.17
	224	0.30	0.75	0.42
	451	0.50	0.63	0.56
*Maximal F-score*	555	0.57	0.59	0.58
Baseline + Clinical Management + Evidence Marker + Prognosis	61	0.10	0.90	0.17
	230	0.30	0.73	0.42
	568	0.50	0.50	0.50
*Maximal F-score*	479	0.47	0.56	0.51

Genes were ranked based on frequency and evaluated against the Alzheimer’s gold standard. For the different selections recall, precision, F-score and rank has been estimated for 10, 30, and 50% recall. In addition the maximal recall has been estimated.

**Figure 7:**
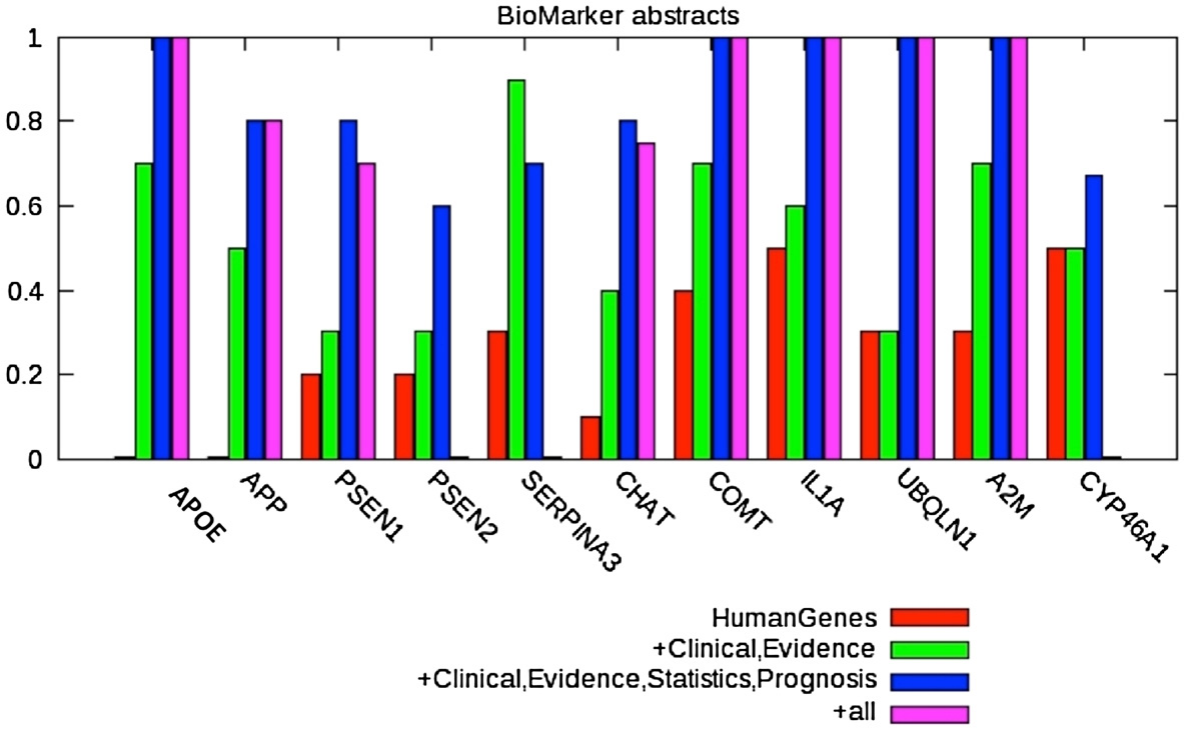
**Proportion of retrieved abstracts containing biomarker information for Alzheimer’s disease.** The red color represents the retrieval of all abstracts containing human genes/proteins. The other color codings indicate the retrieval rate after additive inclusion of further terminology classes.

The analysis of the retrieved abstracts for the first 10 ranked genes implies that the combination of different biomarker classes (*Clinical Management* and *Evidence Marker* alone or together with *Diagnostics* and *Prognosis*) leads to a reduction in the number of abstracts but to an increase in the number of remaining ones (Figure 7). At this point, the user has to decide about the trade-off between recall and precision. For genes with a high number of references (e.g. APP or APOE) a more stringent selection of abstracts is favorable, whereas for genes with a lower number of references a less restrictive search strategy might be beneficial.

### Analysis of novel candidates as potential biomarkers

Genes retrieved by the selection of *Clinical Management* and *Evidence Marker* classes and not mentioned in the AD gold standard were checked manually by a biologist using the SCAIView environment*.* Such genes/proteins might be valuable for identification of novel biomarkers because they represent the yet-to-be identified biomarkers whereas those already matched with the gold standard are candidates that are better known as potential indicators of the disease. For this purpose, the abstracts must contain at least the information of the gene, which is altered (e.g. overexpression or mutation) in a particular state of AD and their therapeutic response state. Examples of such information with the corresponding PMIDs are given in Table 4.

**Table 4:** Examples of articles accepted to contain biomarker information

**PMID**	**Gene**	**Alteration**	**Textual evidence**
17387528	ACAD8 HMGCS2	SNP	In a European screening sample of 115 sporadic AD patients and 191 healthy control subjects, we analyzed single nucleotide polymorphisms in 28 cholesterol-related genes for association with AD. The genes HMGCS2, FDPS, RAFTLIN, ACAD8, NPC2, and ABCG1 were associated with AD at a significance level of P < or = 0.05 in this sample.
17531353	SLC17A7	Protein expression decrease	Loss of VGLUT1 and VGLUT2 in the prefrontal cortex is correlated with cognitive decline in Alzheimer disease…We quantified VGLUT1 and VGLUT2 in the prefrontal dorsolateral cortex (Brodmann area 9) of controls and AD patients using specific antiserums. A dramatic decrease in VGLUT1 and VGLUT2 was observed in AD using Western blot
19863188	HPX SERPINF1	Cerebrospinal fluid concentration	Five differentially-expressed proteins with potential roles in amyloid-beta metabolism and vascular and brain physiology [apolipoprotein A-1 (Apo A-1), cathepsin D (CatD), hemopexin (HPX), transthyretin (TTR), and two pigment epithelium-derived factor (PEDF) isoforms] were identified. Apo A-1, CatD and TTR were significantly reduced in the AD pool sample, while HPX and the PEDF isoforms were increased in AD CSF

Example evidence for genes retrieved via SCAIView for Alzheimer’s and MS diseases but not found in the corresponding gold standards.

For AD, out of 400 genes, 158 genes/proteins had at least one evidence in the literature as being a potential biological indicator of Alzheimer’s disease and thus were considered as true positives (~ 40%) whereas 241 genes/proteins were recognized by the annotator as false positive genes (~ 60%) due to the lack of relevance to either Alzheimer’s disease or biomarkers (Table 5).

**Table 5:** Evaluation of genes not found in AD gold standard but retrieved using the biomarker terminology

**No. of genes/ proteins retrieved by SCAIView but not contained in gold standard**	**No. of genes with at least one biomarker evidence in Medline**	**No. of abstracts with lack of relevance either to the disease or to being a biomarker**
400	158	241

## Discussion

Protein biomarkers are required for informed decision-making in drug discovery and development. In order to evaluate and prioritize potential biomarkers, a systematic literature search is the first step. Abstracts containing information about potential gene biomarkers most often comprise evidence of gene or protein alteration as well as indication words for the clinical investigations. Moreover, diagnostic methods and prognostic terms are found frequently in these abstracts. Automated recognition of gene names, co-occurrence search with disease names and their statistical ranking serve as a baseline for biomarker search.

Using our baseline approach, it turns out that almost all genes/proteins contained in the gold standard were covered by the results of our retrieval system. The system was able to successfully extract biomarker genes/proteins relevant to the queried diseases and both ranking methods performed well for high-ranking genes. Nevertheless, the baseline search returned over 3600 genes from over 33000 abstracts for Alzheimer’s disease and over 2300 genes from more than 12900 abstracts for multiple sclerosis. This amount of abstracts for manual inspection is overwhelming. Making use of the biomarker retrieval terminology developed here results in the significant reduction of the number of extracted genes and documents with almost no loss in the gene enrichment, especially for high-ranking genes. This indicates that inclusion of more biomarker classes in the search query helps to narrow down the retrieval results by adding more restrictive context to the search.

Concerning the statistical ranking of the result set, the frequency-based ranking method performs better than the relative entropy-based ranking for biomarker gene enrichment of the diseases investigated in this study. One explanation is that the relative entropy penalizes genes/proteins common to many investigations/publications and these entities rank at the end of the relevancy list, although they show a high frequency for the disease in question. Another explanation might lay in the selective annotation of entities by annotators of the external AD and MS gold standards; if annotators have selected the most frequent genes for annotation, the gold standard has a bias towards frequency ranking.

Evaluation of retrieved genes not existing in the gold standard for AD showed that almost half of these genes have probably the potential of being considered as potential biomarkers. This indicates that automated text mining using the biomarker terminology combinations increases recall for biomarker information retrieval and makes it possible to explore the biomarker space efficiently.

Comparing the number of retrieved entities between AD and MS shows that the biomarker information for each specific disease is distributed differently over the literature corpora. Thus, to obtain the optimum results, the search strategy should be adapted to each disease of interest; however, combining more general queries with the *Statistics* and/or *Prognosis* classes results in more stringent outputs in terms of biomarker specificity, sensitivity, and predictive parameters. For the purpose of new biomarker identification, it is more suitable to use less stringent combinations, which results in higher recall. In this case, the relative entropy-based ranking method can increase the likelihood of finding proper evidence in the support of a novel biomarker. For new disease areas, it is necessary to update the terminology especially for the class *Diagnostics* which shows the highest diversity of terminology in different disease areas. One possibility to improve the generalisation of the terminology would be to include all diagnostic classes from publicly available terminology resources. However, this might lead to a decline in retrieval precision. Additionally, some medical diagnosis scores are very specific and often do not occur in the large publicly available resources such as MeSH and UMLS.

The flexibility of the biomarker terminology in relation to different query formulations and different biomarker classes should be tested in future investigations.

## Conclusions

The work presented in this paper is a first step towards developing a search engine for literature-based retrieval and in-silico validation of biomarker candidates. It was shown that the development and application of a dedicated biomarker terminology could enhance the retrieval performance significantly through combined search for genes and selected classes of the biomarker retrieval terminology. The experimental results, obtained in the course of this study, demonstrate the effectiveness of the proposed approach that provides a foundation in semantic indexing and retrieval.

## Abbreviations

MeSH: Medical Subject Headings; NER: Names Entity Recognition; OMIM: Online Mendelian Inheritance in Man; UMLS: Unified Medical Language System; NSCLC: Non-Small-Cell Lung Carcinoma; AD: Alzheimer’s Disease; MS: Multiple Sclerosis; PMID: PubMed Identifier.

## Competing interests

The authors declare that they have no competing interests.

## Authors’ contributions

EY carried out text annotations, participated in the terminology design, contributed to evaluations and drafted the manuscript. LT contributed to the study conception and design as well as data acquisition and analysis. BM carried out statistical analyses and maintained the softwares. CMF revised the results critically. NN participated in the design of the study, coordinated the expert annotations, and provided the use case scenario. AS conceived of the study. MHA participated in the design of the study, verified the terminology and revised the paper critically. JF participated in the design of the study and analysis of the results, coordinated the study, participated in evaluations and helped to draft the manuscript. All authors read and approved the final manuscript.

## Pre-publication history

The pre-publication history for this paper can be accessed here:

http://www.biomedcentral.com/1472-6947/12/148/prepub

## Supplementary Material

Additional file 1 List of the PMIDs of the abstracts used for evaluation of the system. Click here for file

## References

[B1] Ghosh D Poisson LM Omics data and levels of evidence for biomarker discovery Genomics 2009 93 13 16 10.1016/j.ygeno.2008.07.006 18723089

[B2] Group BDW Biomarkers and surrogate endpoints: preferred definitions and conceptual framework Clin Pharmacol Ther 2001 69 89 95 11240971 10.1067/mcp.2001.113989

[B3] Perera FP Weinstein IB Molecular epidemiology: recent advances and future directions Carcinogenesis 2000 21 517 524 10.1093/carcin/21.3.517 10688872

[B4] Mayeux R Biomarkers: potential uses and limitations NeuroRx 2004 1 182 188 10.1602/neurorx.1.2.182 15717018 PMC534923

[B5] Timbrell J Types of biomarker and challenges for new biomarkers Toxicol Lett 2006 164 Suppl 1 S315

[B6] Altar CA The biomarkers consortium: on the critical path of drug discovery Clin Pharmacol Ther 2008 83 361 364 10.1038/sj.clpt.6100471 18183037

[B7] Wagner JA Strategic approach to fit-for-purpose biomarkers in drug development Annu Rev Pharmacol Toxicol 2008 48 631 651 10.1146/annurev.pharmtox.48.113006.094611 17937595

[B8] Marrer E Dieterle F Impact of biomarker development on drug safety assessment Toxicol Appl Pharmacol 2010 243 167 179 10.1016/j.taap.2009.12.015 20036272

[B9] Bakhtiar R Biomarkers in drug discovery and development J Pharmacol Toxicol Methods 2008 57 85 91 10.1016/j.vascn.2007.10.002 18024093

[B10] Hurko H Jones GK Valuation of biomarkers Nat Rev Drug Discov 2011 10 253 254 10.1038/nrd3417 21455229

[B11] Ongenaert M Dehaspe L Integrating automated literature searches and text mining in biomarker discovery BMC Bioinforma 2010 11 Suppl 5 O5 10.1186/1471-2105-11-S5-O5

[B12] Krallinger M Valencia A Text-mining and information-retrieval services for molecular biology Genome Biol 2005 6 224 10.1186/gb-2005-6-7-224 15998455 PMC1175978

[B13] Harsha HC Kandasamy K Ranganathan P Rani S Ramabadran S Gollapudi S Balakrishnan L Dwivedi SB Telikicherla D Selvan LDN Goel R Mathivanan S Marimuthu R DeCaprio JA Srivastava S Hanash SM Htuban RH Pandey A A compendium of potential biomarkers of pancreatic cancer PLoS Med 2009 6 e1000046 10.1371/journal.pmed.1000046 19360088 PMC2661257

[B14] BioCreAtIvE workshop[http://www.biocreative.org]

[B15] Blaschke C Hirschman L Valencia A Yeh A A critical assessment of text mining methods in molecular biology BMC Bioinforma 2004 6 Suppl 1 S1 S23 16468169

[B16] Hirschman L Krallinger M Wilbur J Valencia A The BioCreAtIvE II - critical assessment for information extraction in biology challenge Genome Biol 2008 9 Suppl 2 S1 S14 10.1186/gb-2008-9-s2-s1 18834487 PMC2559980

[B17] Pennings JL Koster MP Rodenburg W Schielen PC de Vries A Discovery of novel serum biomarkers for prenatal down syndrome screening by integrative data mining PLoS One 2009 4 e8010 10.1371/journal.pone.0008010 19956656 PMC2777317

[B18] Deng X Geng H Bastola DR Ali HH Link test–a statistical method for finding prostate cancer biomarkers Comput Biol Chem 2006 30 425 433 10.1016/j.compbiolchem.2006.09.002 17126079 PMC1941704

[B19] Bundschus M Dejori M Stetter M Tresp V Kriegel HP Extraction of semantic biomedical relations from text using conditional random fields BMC Bioinforma 2008 9 207 10.1186/1471-2105-9-207 PMC2386138 18433469

[B20] Elkin PL Tuttle MS Trusko BE Brown HB BioProspecting: novel marker discovery obtained by mining the bibleome BMC Bioinforma 2009 10 Suppl 2 S9 10.1186/1471-2105-10-S2-S9 PMC2646243 19208197

[B21] Islam MT Shaikh M Nayak A Ranganathan S Mishra BK, Kekre HB, Thampi GT, Gharpure P, Mukherji A, Lohani RB Biomarker Information Extraction Tool (BIET) development using natural language processing and machine learning Proceedings of the International Conference and Workshop on Emerging Trends in Technology: 26–27 February 2010 2010 ICWET, Mumbai 121 126

[B22] Friedrich CM Dach H Gattermayer T Engelbrecht G Benkner S Hofmann-Apitius M Solomonides T, Silverstein JC, Saltz J, Legre Y, Kratz M, Foster I, Breton V, Beck JR @neuLink: a service-oriented application for biomedical knowledge discovery Proceedings of HealthGrid 2008; 2–4 June 2008 2008 IOS Press, Chicago 165 172 18560118

[B23] Benkner S Arbona A Berti G Chiarini A Dunlop R Engelbrecht G Frangi AF Friedrich CM Hanser S Hasselmeyer P Hose RD Iavindrasana J Köhler M Iacono LL Lonsdale G Meyer R Moore B Rajasekaran H Summers PE Wöhrer A Woods S @neurIST: infrastructure for advanced disease management through integration of heterogeneous data, computing, and complex processing services IEEE Trans Inf Technol Biomed 2010 14 1365 1377 20435543 10.1109/TITB.2010.2049268

[B24] Hanisch D Fluck J Mevissen HT Zimmer R Playing biology's name game: identifying protein names in scientific text Pac Symp Biocomput 2003 8 403 14 12603045

[B25] Hanisch D Fundel K Mevissen HT Zimmer R Fluck J ProMiner: rule based protein and gene entity recognition BMC Bioinforma 2005 6 Suppl 1 S14 10.1186/1471-2105-6-S1-S14 PMC1869006 15960826

[B26] Morgan AA Lu Z Wang X Cohen AM Fluck J Ruch P Divoli A Fundel K Leaman R Hakenberg J Sun C Liu HH Torres R Krauthammer M Lau WW Liu H Hsu CN Schuemie M Cohen KB Hirschman L Overview of BioCreAtIvE II gene normalization Genome Biol 2008 9 S3 18834494 10.1186/gb-2008-9-s2-s3 PMC2559987

[B27] Kullback S Leibler R On information and sufficiency Ann Math Stat 1951 22 79 86 10.1214/aoms/1177729694

[B28] Büttcher S Clarke CLA Cormack GV Information retrieval: implementing and evaluating search engines Cambridge, Mass. MIT Press 296 298

[B29] Smeeton NC Early history of the kappa statistic Biometrics 1985 41 795

[B30] BIOBASE BKL Proteome database[http://www.biobaseinternational.com/index.php?id=proteomedatabases]

[B31] Subramanian A Tamayo P Mootha VK Mukherjee S Ebert BL Gillette MA Paulovich A Pomeroy SL Gloub TR Lander ES Mesirov JP Gene set enrichment analysis: a knowledge-based approach for interpreting genome-wide expression profiles Proc Natl Acad Sci 2005 102 15545 15550 10.1073/pnas.0506580102 16199517 PMC1239896

[B32] Goutte C Gaussier E A probabilistic interpretation of precision, recall and F-score, with implication for evaluation Advances in Information Retrieval. Lecture Notes in Computer Science 2005 3408 345 59

[B33] Szabo E Driscoll B MUC1 expression in lung cancer Lung Cancer, Methods in Molecular Medicine 2003 3 Humana Press, New Jersey 251 258 Volume 74 10.1385/1-59259-323-2:251 12415700

[B34] Petty RD Nicolson MC Kerr KM Collie-Duguid E Murray GI Gene expression profiling in non-small cell lung cancer, from molecular mechanisms to clinical application Clin Cancer Res 2004 10 3237 10.1158/1078-0432.CCR-03-0503 15161676

